# Brain and art

**DOI:** 10.3389/fnhum.2014.00465

**Published:** 2014-06-27

**Authors:** Idan Segev, Luis M. Martinez, Robert J. Zatorre

**Affiliations:** ^1^Department of Neurobiology and The Edmond and Lily Safra Center for Brain Sciences, The Hebrew University of JerusalemJerusalem, Israel; ^2^Spanish National Research Council, Instituto de Neurociencias de AlicanteSant Joan d'Alacant, Spain; ^3^Montreal Neurological Institute, McGill UniversityMontreal, QC, Canada

**Keywords:** neuroesthetics, creativity, performing-arts, emotion, perception

## Introduction

Museums, concerts, dance performances and films attract billions of people worldwide. Indeed, visual art and music have been with us essentially from the beginning of our species. This must mean that Art (and hence, artists) succeed to tap into particular and powerful mechanisms in our brain. This artistic success also means that understanding the phenomenon of Art is a key challenge for modern brain research.

Could we understand, in biological terms, the unique and fantastic capabilities of the human brain to both create and enjoy art? In the past decade neuroscience has made a huge leap in developing experimental techniques as well as theoretical frameworks for studying emergent properties following the activity of large neuronal networks. These methods, including MEG, fMRI, sophisticated data analysis approaches and behavioral methods, are increasingly being used in many labs worldwide, with the goal to explore brain mechanisms corresponding to the artistic experience.

The 37 articles composing this unique *Frontiers Research Topic* bring together experimental and theoretical research, linking state-of-the-art knowledge about the brain with the phenomena of Art. It covers a broad scope of topics, contributed by world-renowned experts in vision, audition, somato-sensation, movement, and cinema. Importantly, as we felt that a dialog among artists and scientists is essential and fruitful, we invited a few artists to contribute their insights, as well as their art.

In this context, we would like to highlight a key similarity between artists and scientists, in particular neuroscientists. Both art and science seek to explain—each with their own unique set of concepts and tools—the *unknown*. Both science and art often claim to seek the “truth.” The focus of modern brain research is to unravel the physical basis that underlies the emerging capabilities of the brain—perception, behavior, emotions and brain-related diseases, whereas the arts elaborate intricately and persistently on these brain-related properties including diseases (see several papers in this volume on art and brain damage) exploring, and in this process also expanding, the range of brain's perceptual and emotional capacity.

This is a unique forte of the arts, which utilize the powerful capacity of the brain to adaptively (plastically) change following perception and action, and propose new ways to view and interpret the world. Indeed, as highlighted in the present volume, art may invoke new “brain states” that are otherwise less likely to be activated by our day-to-day “reality.” Art therefore serves to explore and expand the potential capacity of the brain, e.g., via the recent invention of abstract art (see this volume).

In that sense, brain research and the arts are closely interlinked; modern brain researchers have much new to say about the phenomenon of art from a neuro-scientific perspective (hence the new term “Neuroesthetics” coined in by Semir Zeki), whereas artists have a lot to say about how the brain (they typically use the term “mind,” as do cognitive psychologists) dealing with what it perceives (consciously or unconsciously). This artistic exploration of the mind is well summarized in the surrealistic manifesto by André Breton who said “*It is by pure psychic automatism by which one intends to express verbally, in writing or by other method, the real functioning of the mind. Dictation by thoughts without any control exercised by reason, and beyond aesthetics or moral preoccupation*.”

Joan Miró said that “*art is the search for the alphabet of the mind*.” This volume reflects the state of the art search to understand the neurobiological alphabet of the Arts. We hope that the wide range of articles in this volume will be highly attractive to brain researchers, artists and the community at large.

### Conflict of interest statement

The authors declare that the research was conducted in the absence of any commercial or financial relationships that could be construed as a potential conflict of interest.

